# Transverse oscillations and an energy source in a strongly magnetized sunspot

**DOI:** 10.1038/s41550-023-01973-3

**Published:** 2023-05-25

**Authors:** Ding Yuan, Libo Fu, Wenda Cao, Błażej Kuźma, Michaël Geeraerts, Juan C. Trelles Arjona, Kris Murawski, Tom Van Doorsselaere, Abhishek K. Srivastava, Yuhu Miao, Song Feng, Xueshang Feng, Carlos Quintero Noda, Basilio Ruiz Cobo, Jiangtao Su

**Affiliations:** 1 Institute of Space Science and Applied Technology, Harbin Institute of Technology, Shenzhen, Guangdong China; 2 Key Laboratory of Solar Activity and Space Weather, National Space Science Center, Chinese Academy of Sciences, Beijing, China; 3 Shenzhen Key Laboratory of Numerical Prediction for Space Storm, Harbin Institute of Technology, Shenzhen, Guangdong China; 4 Big Bear Solar Observatory, New Jersey Institute of Technology, Big Bear City, CA USA; 5 Center for Solar-Terrestrial Research, New Jersey Institute of Technology, Newark, NJ USA; 6 Centre for mathematical Plasma Astrophysics, Mathematics Department, KU Leuven, Leuven, Belgium; 7 Instituto de Astrofísica de Canarias (IAC), San Cristóbal de La Laguna, Tenerife Spain; 8 Dept. Astrofísica, Universidad de La Laguna, San Cristóbal de La Laguna, Tenerife Spain; 9 Institute of Physics, University of M. Curie-Skłodowska, Lublin, Poland; 10 Department of Physics, Indian Institute of Technology (BHU), Varanasi, India; 11 School of Information and Communication, Shenzhen Institute of Information Technology, Shenzhen, Guangdong China; 12 Faculty of Information Engineering and Automation, Kunming University of Science and Technology, Kunming, Yunnan China; 13 National Astronomical Observatories, Chinese Academy of Sciences, Beijing, China; 14 School of Astronomy and Space Sciences, University of Chinese Academy of Sciences, Beijing, China

**Keywords:** Solar physics, Solar physics

## Abstract

The solar corona is two to three orders of magnitude hotter than the underlying photosphere, and the energy loss of coronal plasma is extremely strong, requiring a heating flux of over 1,000 W m^*−*2^ to maintain its high temperature. Using the 1.6 m Goode Solar Telescope, we report a detection of ubiquitous and persistent transverse waves in umbral fibrils in the chromosphere of a strongly magnetized sunspot. The energy flux carried by these waves was estimated to be 7.52 × 10^6^ W m^−2^, three to four orders of magnitude stronger than the energy loss rate of plasma in active regions. Two-fluid magnetohydrodynamic simulations reproduced the high-resolution observations and showed that these waves dissipate significant energy, which is vital for coronal heating. Such transverse oscillations and the associated strong energy flux may exist in a variety of magnetized regions on the Sun, and could be the observational target of next-generation solar telescopes.

## Abstract (web summary)

High-resolution observations reveal fibril motions in the chromospheric umbra of a sunspot, providing a potential energy source for coronal heating.

## Main

The Sun’s atmosphere consists of ionized plasma in a magnetic field, and the exchange of energy occurs there at a variety of time and length scales. The reorganization of the magnetic field, known as magnetic reconnection, could generate flows of energy and plasma, so it is considered to be the main mechanism of plasma heating and solar eruptions^1–6^. As the magnetic field is anchored deep inside the solar atmosphere, it is laden with ionized plasma and could act as an effective waveguide for magnetohydrodynamic (MHD) waves (for example, Alfvén, fast and slow magnetoacoustic modes). These MHD waves have potential to carry a substantial amount of energy for plasma heating^7–13^.

Slow magnetoacoustic waves are ubiquitously detected in sunspots and open coronal loops; however, their energy fluxes are relatively low for plasma heating. Fast magnetoacoustic waves carry comparatively stronger energy fluxes, but the occurrence rate is too low to maintain the plasma temperature at millions of kelvin^13–16^. Alfvén waves carry a strong energy flux and could dissipate the energy with high efficiency. However, as Alfvén waves do not perturb the plasma density, they cannot be directly observed with optical instruments^7,8,17^. The identification of a strong, persistent and ubiquitous energy source that could carry a sufficient energy flux for plasma heating would represent a milestone in research into coronal heating, but such a compelling observation is beyond the resolving capability of most of the solar telescopes in operation.

How such wave energy transport and dissipation occur in the highly structured solar atmosphere is not yet known^7–13,18,19^. The physical behaviour of the lower solar atmosphere severely affects our understanding of wave propagation and dissipation^12^. However, there may be some observational constraints that hide a significant portion of wave energy as ‘dark energy’^20,21^.

This study aims to detect transverse motion and the associated energy flux in a strongly magnetized sunspot with high-resolution observations and to justify this scenario with state-of-the-art numerical simulations. We first present observations of transverse fibril oscillations in a sunspot and the numerical simulations and mathematical analysis used to estimate the energy flux and transportation of these waves. Then we discuss the characteristics of these transverse waves and their contribution to coronal heating, and summarize the significance of this finding.

## Results

**Fig. 1:**
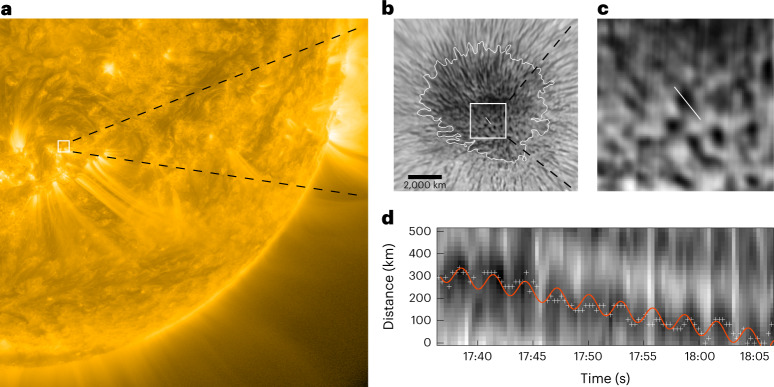
Oscillatory transverse motion of the dark fibril within the sunspot. **a**, Atmospheric Imaging Assembly 171 Å image showing AR12384 on 14 July 2015 at 17:36:22 ut. **b**,**c**, Hα −0.20 Å images showing the sunspot in AR12384 highlighted in **a**. The close-up in **c** highlights the dark fibrils and the slit used to track inherent transverse motions. **d**, Time–distance plot revealing the transverse oscillations (amplitude 39.5 km, phase 0.036). The plus signs mark the fibril’s position and the red curve is the fit from a sinusoidal function and a linear trend. See Supplementary Video 1.

The Visible Imaging Spectrometer installed at the Goode Solar Telescope (GST) at Big Bear Solar Observatory^22,23^ performed high-resolution observations on sunspot AR12384 (Fig. 1 and Supplementary Video 1). The observations started at 17:36:26 ut and stopped at 18:06:26 ut on 14 July 2015. The sampling interval was about 19 s, whereas the camera’s pixel size was 0.029 arcsec (~22 km). The sunspot region was scanned by the Visible Imaging Spectrometer tuned to the Hα 6,563 Å line. The scanning spanned the blue wing (−1.0 Å) to the red wing (1.0 Å) at 0.2 Å intervals. Each image was reconstructed using the Speckle imaging techniques^24^. This method removed pointing errors introduced by turbulence in the Earth’s atmosphere and thus rendered the observational dataset suitable for capturing fine-scale plasma dynamics at sub-arcsecond spatial scales (Fig. 1b,c).

Within the sunspot’s umbra, numerous dark fibrils aligned with nearly vertical magnetic field lines were clearly detected in the chromosphere. They usually had irregular and compact shapes and occupied ~29–33% of the umbral area (Fig. 1). The fibrils had greater densities and elevated formation heights^25^. We estimated that the fibrils formed at heights of about 1,100 km, the lower limit of the formation height of the Hα line in the umbra^25–27^. The dark fibrils might be connected with small-scale magnetic field components in the vertical umbral field observed with the Hinode Caii line^28^. Their brightnesses are enhanced when an umbral flash occurs, suggesting an unresolved plasma heating mechanism. This interaction might offer a clue to the nature of the fibrils and umbral flashes and could be used for seismological applications^29,30^. To maintain the balance of the total pressure (the sum of magnetic and gas pressures), the fibrils should have a lower temperature, and could therefore be the coldest structures detected on the Sun (Fig. 1c). They were detectable in several bandpasses: Hα line core, Hα ±0.2 Å and Hα ±0.4 Å. We only used the Hα −0.2 Å dataset for further analysis in this Article.

The umbras of sunspots have a strongly vertical magnetic component of about 3,000–5,000 G at the photosphere^31^. The magnetic pressure in a sunspot is much greater than the gas pressure, and plasma motions therefore do not drive lateral motion of the magnetic field. Nevertheless, sunspots are optimal waveguides for the slow-mode MHD waves, which perturb plasma motions along the magnetic field lines^32^ but do not transport a sufficiently strong energy flux to the overlying atmosphere^13^.

In this study, we observed transverse oscillations across magnetic field lines in the chromospheric umbra of sunspot AR12384. This spot had a photospheric magnetic field strength of up to 4,000 G, but the field strength at the chromospheric height at which the fibril oscillations were detected was estimated to be 2,300 G (by accounting for the horizontal variation and height stratification^31^; Methods). We placed an artificial slit following an oscillating fibril (Fig. 1c), and traced its lateral motions. The emission intensity along the slit across the fibril was stacked in order of collapsing time to produce a time–distance map. The transverse motion was tracked with an automatic algorithm by locating the minimum emission intensity along the slit, and the uncertainty was assumed to be half of the diffraction-limited resolution of the GST at 6,563 Å (or 32 km). The transverse motion was subsequently fitted with a sinusoidal function and a linear trend to obtain the oscillation amplitude and period (Methods). The fibril presented in Fig. 1 as an example oscillated with a period of 173 ± 1.98 s and a displacement amplitude of 39.5 ± 4.48 km. We analysed the transverse oscillation of eight sample fibrils (Supplementary Table 1). The average oscillation period was 239 ± 48.6 s and the average displacement amplitude was 49.5 ± 8.75 km. In the time–distance map we identified clear standing umbral oscillations and umbral flashes (that is, recurring vertical stripes with enhanced emission intensity), both with a period of ~3 min. With the high-quality GST observations, we were able to discern collective periodic perturbation across the whole umbra (Supplementary Video 1). This type of motion may be linked to MHD eigenmodes of sunspots^33^. Fibril oscillations have a period of about 4 min, which is significantly longer than that of umbral oscillations; this difference in the periodicity implies a distinctive nature of the transverse fibril oscillations.

These fibril oscillations had a significant transverse displacement perpendicular to the magnetic field of the umbra. This is a feature of kink waves trapped within a magnetic flux tube^34,35^. This Article reports observations of kink waves in a kilogauss magnetized sunspot umbra. Such transverse waves are traditionally observed in coronal loops, which have magnetic field strengths of about 10–100 G and densities that are 4–6 orders of magnitude smaller. The energy flux carried by these kink waves in coronal loops is not sufficient to compensate the energy loss^13^. Transverse oscillation has been detected before in fibrils at the edge of a sunspot^36^ or the superpenumbra^37^. Our case is distinct from these studies. First, the magnetic field strength at the edge or the superpenumbra of a sunspot is about 100 G, whereas in the sunspot of interest here the magnetic field strength reached 2,300 G at the chromospheric umbra. This means that to perturb the magnetic field laterally, the pressure gradient or Lorentz force (or a combination of both) must be at least 500 times stronger. Second, at the edge or superpenumbra of a sunspot, the influence of other polarities becomes strong and complex, and would trigger a number of magnetic and fluid dynamics. In contrast, the umbra of a sunspot has a nearly vertical magnetic field, effectively suppressing lateral fluid motions. Our study implies that other strong and persistent dynamics at smaller scales could be found in the umbra. Third, as a result of the high spatial resolution and sensitivity of the GST, it is now possible to observe a kink MHD mode by directly tracking features inside the umbra. This opens a route to a series of diagnostic applications in sunspots, as well as offering a mechanism for coronal heating, which could be important targets for next-generation telescopes with better resolution and sensitivity, such as the European Solar Telescope and Daniel K. Inouye Solar Telescope.

An intriguing aspect of these observations is that the transverse oscillations were detected in colder and denser magnetic flux tubes rooted in the kilogauss sunspot umbra. No significant damping was detected within these transverse oscillations, which implies that these waves were continuously driven by a periodic source in the umbra. The transverse waves detected in this study could perturb the magnetic field to a non-negligible extent on the order of a few hundred gauss, and must therefore carry a substantial amount of energy to the upper atmosphere. These waves continuously pump energy from the sunspot to the plasma confined in overlying large-scale coronal loops. In this scenario, we suggest that active-region coronal loops oscillate persistently with larger amplitudes, as their magnetic field strengths and densities are much lower than those of the underlying sunspot. In reality, however, either the coronal loops oscillate only after solar flares and are damped after a few oscillation cycles^38,39^ or the oscillations have very small amplitudes, eventually known as decay-less transverse oscillations, and are detected only after amplification of the motion^40^. Therefore, we infer that in the present case the wave energy flux is either dissipated or reflected by the inhomogeneity of the magnetic field and density within this sunspot. In either case, the persistent transverse oscillations were kink waves propagating in fibrils and carried a strong energy flux, which must be released in the form of solar wind or nanoflares, subsequently causing dynamical plasma processes and heating.

**Fig. 2:**
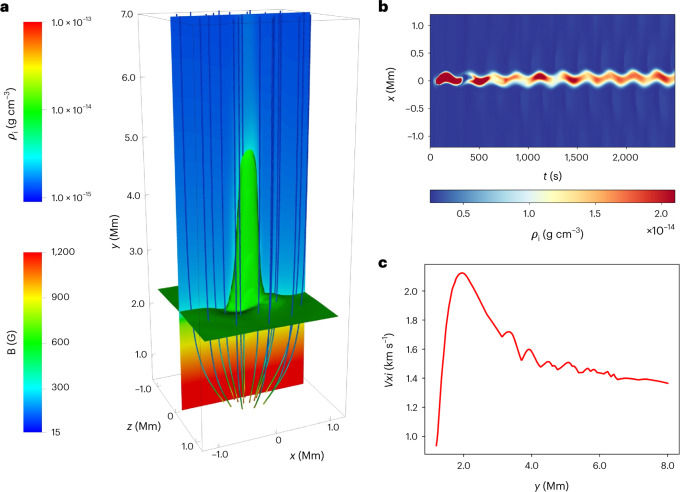
Numerical simulation of driven propagating fast magnetoacoustic wave. **a**, 3D structure of the magnetic field (**B**) lines, the stratified sunspot atmosphere in terms of ion mass density (*ρ*_i_) and the initial simulated fibril structure. **b**, A horizontal cut at the fibril centre revealing the transverse motion. **c**, The amplitude of the oscillations in the ion velocity as a function of height. See Supplementary Video 2.

To demonstrate the propagation of kink waves in magnetic fibrils and the associated energy transportation, we performed a 3D two-fluid simulation of sunspot dynamics. The numerical sunspot atmosphere included the photosphere, chromosphere, transition region and corona, and was permeated by a diverging kilogauss magnetic field (Fig. 2a and Methods). To generate a cold and dense umbral fibril, we added a slender vertical region with enhanced pressure *p* in the ambient equilibrium atmosphere, which is given by:

1 $${p}_{\alpha }={p}_{0\alpha }\left(1+{A}_{\mathrm{p}}\exp \left(-\frac{{x}^{2}+{(y-{y}_{0})}^{2}+{z}^{2}}{{w}^{2}}\right)\right)$$

where *α* represents either ions (i) or neutral species (n) and *A*_p_ is the pulse amplitude, *p*_0__α_ is the gas pressure of ions or neutral species in the equlibrium atmosphere, *x*, *y*, and z are the coordinates used in the simulation domain, and the parameters *y*_0_ and *w* control the height and width of the numerical fibril. A periodic transverse driver was operating at the bottom boundary of the model fibril:

2 $${v}_{{x}_{\alpha }}={A}_{{v}_{x}}\exp \left(-\frac{{x}^{2}+{(y-{y}_{0})}^{2}+{z}^{2}}{{w}^{2}}\right)\sin \left(\frac{2\pi }{{P}_{d}}t\right)$$

where *t* is time. This driver could generate an upwardly propagating kink wave. The parameters were set up to mimic the average observational values: *A*_p_ = 100, velocity perturbation $A_{v_x}=5\,{\mathrm{km}}\,{\mathrm{s}}^{-1}$, width of pulse *w* = 50 km and driver period *P*_d_ = 240 s.

The length of the fibril generated in the simulation varied between 2,000 km and 5,000 km with an average width of about 100 km (Fig. 2a), and exhibited a periodic transverse displacement (Fig. 2b). This periodic motion is a pattern of trapped fast waves in the magnetic flux tube (the fibril). This kink wave was driven by the periodic lateral displacement at the bottom boundary, and was guided by the fibril structure—a denser region with a slower Alfvén speed. This fibril oscillated with a period of 240 s. The displacement amplitude of this guided kink wave varied with the height, but closely matched the observed values (about 50 km). Figure 2c plots the ion velocity amplitude of the kink wave as a function of height. It clearly shows that the velocity amplitude was about 1.0 km s^−1^ at the middle of the chromosphere (*y* = 1.0 Mm). It increased with height and reached a maximum value (~2.1 km s^−1^) at a height of *y* = 2.1 Mm at the top of the chromosphere. The velocity amplitude began to drop thereafter to ~1.4 km s^−1^ as the wave reached the transition region and corona (*y* *>* 2.1 Mm). The velocity amplitude was consistent with the observed average amplitude of ~1.4 km s^−1^ (Supplementary Table 1). The initial growth of the wave amplitude was associated with a density decrease with height, and this trend competed with the energy dissipation caused by ion–neutral collisions in the chromosphere^41^. Some of the kink wave energy leaked laterally to the ambient plasma in the chromosphere and transition region, and a proportion is transmitted to the inner corona (Fig. 2b and Supplementary Video 2).

**Fig. 3:**
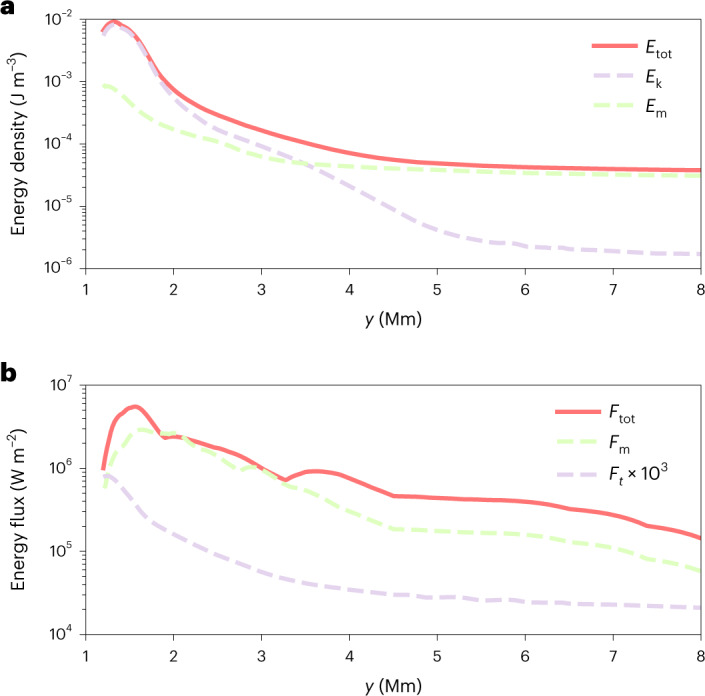
Energy densities and fluxes carried by the fast kink wave in the simulation. **a**, Kinetic (*E*_k_), magnetic (*E*_m_) and total (*E*_tot_) energy densities as a function of height. **b**, Thermal (*F*_t_), magnetic (*F*_m_) and total (*F*_tot_) energy fluxes in the oscillating fibril. The thermal energy flux was amplified by a factor of 1,000 for visualization purposes.

Our observations and two-fluid MHD simulation reveal that fast kink waves are excited in the lower atmosphere of sunspots, and cause transverse displacement of umbral fibrils in a strong magnetic field environment. The energy carried by these MHD waves could act as an energy reservoir and trigger many other energetic processes in sunspots and the overlaying active regions. We then estimated the total energy flux carried by these fast kink waves. As direct measurement of the plasma parameters is not possible with modern instrumentation, we took the Stokes measurement made by the Near-Infrared Imaging Spectropolarimeter at the Big Bear Solar Observatory and used Stokes inversion based on response functions code to calculate magnetic field, plasma density and temperature for the various optical depths (Methods). A fast kink wave model in a magnetized plasma cylinder was developed (Methods) to calculate the energy flux. The energy flux was calculated by integrating over the magnetic flux and the ambient plasma and averaged over one wave cycle, and the filling factor was set to 0.31 in accordance with the fraction of the umbral area filled with fibrils. The total energy flux along the vertical direction in a chromospheric magnetic environment of about 2,300 G was estimated to be about 7.5 × 10^6^ W m^−2^; this component transfers energy upwards and acts as a plasma heating flux. The fractions of the Poynting (magnetic) and thermal energy fluxes are listed in Supplementary Table 3. To assess the consistency between the high-resolution observation and the numerical simulation, Fig. 3 presents the energy density and energy flux in the oscillatory fibril in the simulation; the estimated peak energy flux reaches 6.6 × 10^6^ W m^−2^ at *y* = 1.6 Mm and drops to about 2 × 10^5^ W m^−2^ at *y* = 8 Mm. We could see that despite the numerical model being simplified in terms of both the magnetic field structuring and other non-ideal MHD effects (such as the heating term, radiative loss, thermal conduction and so on), the energy flux reproduced in the simulation is still consistent with the value obtained in the observations within one order of magnitude, and this value is still about two orders of magnitude greater than the radiative loss of the active region. With the assistance of a numerical experiment and a mathematical model, we obtained a very strong energy flux directed to the solar corona, which could play an important role in coronal heating.

## Discussion

In this study we used high-resolution Goode Solar Telescope observations to show that dark fibrils within a sunspot’s umbra oscillated transversely across a strong (kilogauss) magnetic field. These transverse oscillations are basically fast kink waves trapped within vertically elongated fibrils. We identified an important energy source in a strongly magnetized sunspot using high-resolution observations. To calculate the energy density and flux carried by these waves, we developed an analytical model for a plasma cylinder, and derived formulae for the energy densities and fluxes. The plasma and magnetic field parameters within the sunspot were obtained by Stokes inversion. We found that the energy fluxes carried by these kink waves were three to four orders of magnitude stronger than the required heating rate for plasma in active regions^42^. These fluxes are permanently directed to the upper atmosphere, but the waves were attenuated as they reached the corona—as demonstrated by two-fluid simulations.

The two-fluid MHD modelling shows that the ion–neutral collision term could effectively dissipate a significant amount of propagating kink wave energy into the sunspot plasma. This process is particularly pronounced in moderately ionized layers, such as upper chromosphere. This physical mechanism has also been found to be effective in heating the quiet regions of the solar atmosphere by energy dissipation of acoustic, magnetoacoustic and Alfvén waves^43–45^.

The periodicity of the observed fibril oscillations is another important feature for seismology applications. The fibril oscillated with an average period of about 4 min, significantly distinct from 3 min umbral oscillations, which were believed to be slow-mode MHD waves propagating upwards along the vertical umbral field lines^15^. Although we cannot reveal the origin of the periodicity of the transverse fibril oscillations at this stage, we suggest that it may be triggered by dynamics at smaller length scales that have not been fully resolved by modern solar telescopes. The high-quality data reveal the existence of a global collective motion of the umbra, which might be the MHD eigenmode wave within the sunspot^33^. They may modulate the collective behaviour of the fibrils and provide another approach for seismological applications.

As shown in the two-fluid simulations, the kink MHD waves dissipated in the sunspot atmosphere or leaked laterally. We infer that this may be connected to two other forms of solar atmospheric oscillations: running penumbral waves^26^ and decay-less coronal loop oscillations^40,46^. The running penumbral wave could be a form of wave due to lateral leakage of the fibril oscillations, as the penumbral waves have periods of 4–6 min and are usually detected at the umbra–penumbral boundary. The decay-less oscillations of the active region loops have small amplitudes at sub-pixel scales and have negligible damping rates. Their oscillation periods are also within the range of the fibril oscillations of this study^46^; decay-less oscillations of active region coronal loops could therefore be the residue of attenuated fibril oscillations.

The transverse fibril oscillations at the core of a sunspot represent a strong energy source or reservoir in dark and dynamic features. This type of energy source might also exist in other basic magnetized solar structures at diverse spatiotemporal scales, such as in coronal holes, pores, granules, prominences and open coronal structures. They could generate kink waves with strong energy fluxes and act as a source for solar plasma heating. As the fibrils were found to be ubiquitous at the very core of the sunspot, and occupied about 30% of the umbral area, it is likely that they are the footpoints of high-reaching coronal loops. Many of the wave heating models developed for coronal heating take a simple transverse driver at the coronal base^13^. This is because it is not yet known how loops are driven at their footpoints to generate transverse waves. This study sheds light on how such high-reaching coronal loops may be shaken to generate waves that dissipate higher up in the corona. This type of feature and its fine dynamics could be an important target for 4 m solar telescopes such as the European Solar Telescope and the Daniel K. Inouye Solar Telescope, as they could play important roles in both solar plasma heating and seismological applications.

## Conclusions

In this Article we present the observation of transverse waves of dark fibrils in the chromospheric umbra of a sunspot with photospheric magnetic field strengths of up to 4,000 G with the 1.6 m Goode Solar Telescope, which has the largest aperture of fully operating solar telescopes. We computed the wave energy flux by applying a cutting-edge Stokes inversion method and a stringent analytical model of the kink MHD waves. It was found that the ubiquitous fibril oscillations that perturbed the chromospheric magnetic field at a level of about 2,300 G carried energy fluxes of about 7.52 × 10^6^ W m^−2^, three to four orders of magnitude stronger than the radiative loss of active region plasma. This is a strong and persistent energy source not resolved by other solar telescopes, and could represent a substantial contribution to plasma heating in active regions. We then used a two-fluid MHD model to simulate kink wave excitation and propagation in a strongly magnetized sunspot. The energy density and energy fluxes calculated in the numerical model were found to be consistent with our observations. The high-resolution observations and state-of-the-art MHD simulation thereby justify our interpretation of a scenario in which energy transfer in a sunspot with a strong magnetic field that connects the photosphere, chromosphere and corona acts as an effective channel for transporting the dynamic energy from the sub-photosphere to the upper atmosphere.

## Methods

### Data analysis and kinetic study

#### Data preprocessing

On 14 July 2015 the Goode Solar Telescope observed sunspot AR12384 with the Visible Imaging Spectrometer, which took images with central wavelengths tuned to one of 11 spectral positions between Hα −1.00 Å and H_*α*_ +1.00 Å; the bandpass was set to 0.07 Å. Each image was reconstructed with a burst of 25 short-exposure images using Speckle imaging techniques^24^. This step removed the pointing error introduced by turbulence in the Earth’s atmosphere. The observations started at 17:36:26 ut and lasted for about 30 min. The image sequence had a cadence of about 19 s and a pixel size of 0.029 arcsec. We removed the effect of the telescope rotation and cropped the images into a smaller field-of-view centred on AR12384. The image sequence was then aligned to a sub-pixel accuracy with a cross-correlation technique.

#### Tracking the transverse oscillation

To track the transverse motion of a given dark fibril, we took the emission intensity along a short slit across the fibril and stacked the intensity slice at various times to form a time–distance map (see ref. ^47^ for details). The transverse motion was tracked by automatic detection of the minimal emission intensity along the slit, and the tracking error was assumed to be half of the diffraction-limited resolution of the GST telescope (that is, 0.042 arcsec or 32 km).

The tracked motion was subsequently fitted with a sinusoidal function and a linear trend, which gave the oscillation amplitude and period (Fig. 1). This step was done to track eight fibrils as samples to show the generic behaviour of the fibril oscillations; the results are summarized in Supplementary Table 1.

### Two-fluid MHD modelling of a kink wave in a sunspot

#### Numerical simulation

To simulate a sunspot atmosphere with multiple layers that could contain partially ionized plasma, we considered a two-fluid MHD model that included the dynamics of ions, neutrals and electrons as fluid constituents; the neutrals and ions were coupled by an ion–neutral collisional term. We assumed that hydrogen was the main plasma ingredient and took into account the influence of heavier elements given by the OPAL repository of solar abundances^48^.

The dynamics of mass density, momentum and energy of each constituent fluid in the two-fluid MHD model were described by a set of equations as follows^49^:

3 $$\frac{\partial {\rho }_{{\rm{n}}}}{\partial t}+\nabla \cdot \left(\,{\rho }_{{\rm{n}}}{{\bf{v}}}_{{\rm{n}}}\right)=0,\frac{\partial {\rho }_{{\rm{i}}}}{\partial t}+\nabla \cdot \left(\,{\rho }_{{\rm{i}}}{{\bf{v}}}_{{\rm{i}}}\right)=0$$

4 $${\rho }_{{\rm{n}}}\frac{\partial {{\bf{v}}}_{{\rm{n}}}}{\partial t}+{\rho }_{{\rm{n}}}\left({{\bf{v}}}_{{\rm{n}}}\cdot \nabla \right){{\bf{v}}}_{{\rm{n}}}+\nabla {p}_{{\rm{n}}}-{\rho }_{{\rm{n}}}{\bf{g}}+{\alpha }_{{\rm{in}}}\left({{\bf{v}}}_{{\rm{n}}}-{{\bf{v}}}_{{\rm{i}}}\right)=0$$

5 $${\rho }_{{\rm{i}}}\frac{\partial {{\bf{v}}}_{{\rm{i}}}}{\partial t}+{\rho }_{{\rm{i}}}\left({{\bf{v}}}_{{\rm{i}}}\cdot \nabla \right){{\bf{v}}}_{{\rm{i}}}+\nabla {p}_{{\rm{ie}}}-{\rho }_{{\rm{i}}}{\bf{g}}-{\alpha }_{{\rm{in}}}\left({{\bf{v}}}_{{\rm{n}}}-{{\bf{v}}}_{{\rm{i}}}\right)=\frac{1}{{\mu }_{0}}(\nabla \times {\bf{B}})\times {\bf{B}}$$

6 $$\frac{\partial {E}_{{\rm{n}}}}{\partial t}+\nabla \cdot \left[\left({E}_{{\rm{n}}}+{p}_{{\rm{n}}}\right){{\bf{v}}}_{{\rm{n}}}\right]-{Q}_{{\rm{n}}}-{\rho }_{{\rm{n}}}{\bf{g}}\cdot {{\bf{v}}}_{{\rm{n}}}=0$$

7 $$\frac{\partial {E}_{{\rm{i}}}}{\partial t}+\nabla \cdot \left[\left({E}_{{\rm{i}}}+{p}_{{\rm{ie}}}\right){{\bf{v}}}_{{\rm{i}}}\right]-{Q}_{{\rm{i}}}-{\rho }_{{\rm{i}}}{\bf{g}}\cdot {{\bf{v}}}_{{\rm{i}}}=\nabla \cdot \frac{{\bf{B}}\left({{\bf{v}}}_{{\rm{i}}}\cdot {\bf{B}}\right)}{\mu }$$

8 $$\frac{\partial {\bf{B}}}{\partial t}=\nabla \times \left({{\bf{v}}}_{{\rm{i}}}\times {\bf{B}}\right),\nabla \cdot {\bf{B}}=0.$$

Here *ρ* is the mass density, *p* the gas pressure, **v** the velocity and **B** the magnetic field. The subscripts (n, i or e) label the constituent fluid of neutrals, ions or electrons, or a combination. *µ*_0_ is the vacuum magnetic permeability and ${\bf{g}}=-g\hat{y}$ is the gravitational acceleration vector, the magnitude of which was set at *g* = 274.78 m s^−2^. The total energy densities of neutrals and ions are defined as *E*_n_ = *p*_n_*/*(*γ* − 1) + *ρ*_n_**v**_n_^2^*/*2, *E*_i_ = *p*_ie_*/*(*γ* − 1) + *ρ*_i_**v**_i_^2^*/*2 + **B**^2^*/*2*µ*, where *γ* is the adiabatic index. The collisional terms *Q*_i_ and *Q*_n_, which contain the heating term, *α*_in_(*v*_i_ − *v*_m_)^2^*/*2, are given in refs. ^49–52^. This heating term would result in the conversion of kinetic energy into thermal energy.

A sunspot model was constructed with a stratified atmosphere and a diverging magnetic field. The magnetic field was current-free and axially symmetric about the *y* axis, as given by^53^:

9 $${\bf{B}}(x,y,z)=\left[\frac{-3{Sx}(\,y-y_m)}{{x}^{2}+{(\,y-y_m)}^{2}},\frac{S\left({x}^{2}-2{(\,y-y_m)}^{2}+{z}^{2}\right)}{{x}^{2}+{(\,y-y_m)}^{2}}+{B}_{{\rm{v}}},\frac{-3S(\,y-y_m)z}{{x}^{2}+{(\,y-y_m)}^{2}}\right].$$

The parameter *S* was set by ensuring that the magnetic field strength had maximum value of 1,200 G at the footpoint; *y*_m_ was set to 0.5 and controls the divergence of the magnetic field. The uniform vertical magnetic field component was set at *B*_v_ = 15 G. The magnetic field in use is visualized in Fig. 2.

The initial stratified atmosphere was set to follow the temperature profile of a semi-empirical sunspot model^54^. The ions (electrons) and neutrals were assumed to have equal temperature in the initial equilibrium, and the pressure was given by a stratified atmosphere model:

10 $$\frac{\partial {p}_{\alpha }}{\partial y}=\frac{{\eta }_{\alpha }{m}_{{\rm{H}}}g}{{k}_{{\rm{B}}}}\frac{{p}_{\alpha }}{{T}_{\alpha }}.$$

The reduced atomic masses of neutral and ion + electron were set as *η*_n_ = 1.21 and *η*_i_ = 0.58, respectively. The variations in ion/neutral densities and temperatures are plotted in Supplementary Fig. 1.

#### Initial and boundary conditions

To generate a cold and dense chromospheric fibril, we added a localized pressure profile above the equilibrium condition so that the pressure profile ${p}_{\alpha }^{{{\prime} }}$ was modified to model a fibril:

11 $${p}_{\alpha }^{{\prime} }={p}_{\alpha }\left\{1+{R}_{p}\exp \left[-\frac{{x}^{2}+{(y-{y}_{0})}^{2}+{z}^{2}}{{w}^{2}}\right]\right\}$$

where *p*_*α*_ is the initial stratified pressure. The parameter *w* defines the width of the fibril and was set to 50 km. The relative amplitude *R*_*p*_ was set to 100. A periodic transverse driver was set up at the bottom boundary for every constituent fluid:

12 $${v}_{x,\alpha }={A}_{v}\exp \left[-\frac{{x}^{2}+{(y-{y}_{0})}^{2}+{z}^{2}}{{w}^{2}}\right]\sin \left(\frac{2\pi }{{P}_{\mathrm{d}}}t\right)$$

where *P*_d_ was set to 240 s and the amplitude of the oscillating driver *A*_*v*_ was set to 5 km s^−1^. The parameters were chosen in good agreement with the measurement in this study.

### Observations and inversion of Stokes parameters

#### Stokes observations

The Near Infra-Red Imaging Spectropolarimeter (NIRIS)^55,56^ uses a dual Fabry–Pérot etalon and a state-of-the-art adaptive optical system; it could therefore provide diffraction-limited spectroscopic and polarimetric data over a large field of view from the deep photosphere to the base of the solar corona. The Stokes observations have a field of view of about 85 arcsec and an image scale of 0.083 arcsec per pixel. NIRIS performed full spectropolarimetric measurements at 60 line positions at the Fei (15,648.514 Å) spectral line with a bandpass of 0.1 Å.

#### Stokes inversion method

The propagation of the solar spectrum over a sunspot atmosphere could be described by the radiative transfer equation for polarized light^57^. With spectropolarimetric measurements, one could therefore infer the parameters of the sunspot’s stratified atmosphere, such as the gas density, temperature, velocity, magnetic field vector and so on. In this study, we used the Stokes inversion based on response functions code (SIR^58^) to obtain the plasma parameters at multiple optical depths.

The Stokes inversion was done at the spectral line of 15,648.514 Å, which has a high Landé factor of 3 and is therefore an excellent tracer of magnetic fields. The SIR inversion took an empirical sunspot model (FALC model^59^) as the initial temperature profile, and calculated a stratified atmosphere in hydrostatic equilibrium. The spectral line parameters and elemental abundances were taken from refs. ^60,61^. The SIR inversion gave the plasma temperature, magnetic field vector, line-of-sight velocity and microturbulence and macroturbulence velocities. The gas density was calculated using the equation of state for partially ionized plasma.

We assumed that the velocity parameters were height-independent, and the temperature (and hence the gas density and pressure) and magnetic field were allowed to vary with height. The inversion was repeated five times to minimize the effect of the random initial guess, and the best case was determined by the closest match between the observed and synthesized spectra. The best inversion used in this study is visualized in Supplementary Fig. 2.

### Energy flux estimation of the oscillating fibrils in a sunspot

#### Model of a sunspot fibril and MHD wave

Fibrils are a cooler and denser structure that are elongated along the nearly vertical magnetic field of the sunspot umbra; they are dot shaped or have limited horizontal extents. In this study, we used a plasma cylinder to model umbral fibrils, in which a uniform magnetic field was assumed to be aligned with the axis. The straight cylinder had a radius of *a*. The plasma density and magnetic field were uniform inside and outside the cylinder, and the plasma parameters were discontinuous at the boundary surface. Gravity was neglected, and the background flow was not considered. This plasma cylinder model has been the basis for MHD wave heating and diagnostics in solar physics^13,62^.

The ideal MHD equations were first linearized to separate the equilibrium and perturbed quantities, which are denoted by subscripts 0 and 1 respectively. Details of the model and linearization can be found in refs. ^34,63^. We assumed that any perturbed quantity had the following distribution: $g_{1}(r,\varphi,z,t)=\hat{g}_{1}(r){\mathrm{e}}^{i(m\varphi+{k_{z}}z-\omega{t})}$, where (*r,φ,z*) are the cylindrical coordinates, *m* and *k*_*z*_ are the azimuthal and longitudinal wavenumbers, respectively, and *ω* is the angular frequency; $\hat{g}_{1}(r)$ contains the variation along the radial direction.

The perturbed velocity and magnetic field vectors are given by

13 $${{{\bf{v}}}}_{1}=\left(\begin{array}{c}-\frac{\left({k}_{z}^{2}{v}_{\mathrm{C}}^{2}-{\omega }^{2}\right)\left({v}_{\mathrm{A}}^{2}+{v}_{\mathrm{s}}^{2}\right)}{{\omega }^{2}\left({k}_{z}^{2}{v}_{\mathrm{A}}^{2}-{\omega }^{2}\right)}\frac{{{\mathrm{d}}R}(r)}{{{\mathrm{d}}r}}\\ \frac{-{im}\left({k}_{z}^{2}{v}_{\mathrm{C}}^{2}-{\omega }^{2}\right)\left({v}_{\mathrm{A}}^{2}+{v}_{\mathrm{s}}^{2}\right)}{r{\omega }^{2}\left({k}_{z}^{2}{v}_{\mathrm{A}}^{2}-{\omega }^{2}\right)}R(r)\\ \frac{-i{k}_{z}{v}_{\mathrm{s}}^{2}}{{\omega }^{2}}R(r)\end{array}\right){\mathrm{e}}^{i\left(m\varphi +{k}_{z}z-\omega t\right)}$$

14 $${{\bf{B}}}_{1}=\left(\begin{array}{c}\frac{{k}_{z}{B}_{0z}\left({k}_{z}^{2}{v}_{\mathrm{C}}^{2}-{\omega }^{2}\right)\left({v}_{\mathrm{A}}^{2}+{v}_{\mathrm{s}}^{2}\right)}{{\omega }^{3}\left({k}_{z}^{2}{v}_{\mathrm{A}}^{2}-{\omega }^{2}\right)}\frac{{{\mathrm{d}}R}(r)}{{{\mathrm{d}}r}}\\ \frac{{im}{k}_{z}{B}_{0z}\left({k}_{z}^{2}{v}_{\mathrm{C}}^{2}-{\omega }^{2}\right)\left({v}_{\mathrm{A}}^{2}+{v}_{\mathrm{s}}^{2}\right)}{r{\omega }^{3}\left({k}_{z}^{2}{v}_{\mathrm{A}}^{2}-{\omega }^{2}\right)}\\ \frac{i{B}_{0z}\left({k}_{z}^{2}{v}_{\mathrm{s}}^{2}-{\omega }^{2}\right)}{{\omega }^{3}}R(r)\end{array}\right){\mathrm{e}}^{i\left(m\varphi +{k}_{z}z-\omega t\right)}$$

where ${v}_{\mathrm{A}}={B}_{0z}/\sqrt{{\mu }_{0}{\rho }_{0}}$ is the Alfvén speed, ${v}_{\mathrm{s}}=\sqrt{\gamma {p}_{0}/{\rho }_{0}}$ is the sound speed and ${v}_{\mathrm{C}}={v}_{\mathrm{A}}{v}_{\mathrm{s}}/\sqrt{{v}_{\mathrm{A}}^{2}+{v}_{\mathrm{s}}^{2}}$ is the cusp speed. *ρ*_0_, *p*_0_ and *B*_0*z*_ are the equilibrium density, pressure and magnetic field strength and *γ* = 5*/*3. The quantity *R* is defined by $\nabla\cdot{v}_1=R(r){\mathrm{e}}^{i(m\varphi+k_{s}z-\omega{t})}$. To obtain the eigenmode (body mode) for the MHD wave, *R* should have the following profile^62^:

15 $$R(r)=\left\{\begin{array}{c}{C}_{1}{J}_{m}\left({\widetilde{\kappa }}_{\mathrm{i}}r\right),\text{if}\,r < a\\ {C}_{2}{K}_{m}\left({\kappa }_{\mathrm{e}}r\right),\text{if}\,r > a.\end{array}\right.$$

Here *C*_1_ and *C*_2_ are constants, *J*_*m*_ is the Bessel function of the first kind of order *m*, *K*_*m*_ is the modified Bessel function of the second kind of order *m*, and ${\widetilde{\kappa }}_{\mathrm{i}}=\sqrt{-{\kappa }_{\mathrm{i}}^{2}}$ with *κ*_i_ and *κ*_e_ defined as:

16 $${\kappa }_{\mathrm{i}}=\sqrt{\frac{\left({k}_{z}^{2}{v}_{{\mathrm{Ai}}}^{2}-{\omega }^{2}\right)\left({k}_{z}^{2}{v}_{{\mathrm{si}}}^{2}-{\omega }^{2}\right)}{\left({k}_{z}^{2}{v}_{{\mathrm{Ci}}}^{2}-{\omega }^{2}\right)\left({v}_{{\mathrm{Ai}}}^{2}+{v}_{{\mathrm{si}}}^{2}\right)}},$$

17 $${\kappa }_{\mathrm{e}}=\sqrt{\frac{\left({k}_{z}^{2}{v}_{{\mathrm{Ae}}}^{2}-{\omega }^{2}\right)\left({k}_{z}^{2}{v}_{{\mathrm{se}}}^{2}-{\omega }^{2}\right)}{\left({k}_{z}^{2}{v}_{{\mathrm{Ce}}}^{2}-{\omega }^{2}\right)\left({v}_{{\mathrm{Ae}}}^{2}+{v}_{{\mathrm{se}}}^{2}\right)}}.$$

Here subscripts i and e denote the quantities inside and outside the plasma cylinder. *C*_i_ and *C*_e_ are related by matching the radial component of the perturbed velocity at the boundary surface (*r* = *a*).

#### Energy densities and fluxes carried by a MHD wave

We calculated energy density and fluxes carried by a MHD wave. The total energy density (*e*_tot_) has three constituents: the magnetic (*e*_m_), kinetic (*e*_k_) and internal energy density (*e*_u_), which were averaged over one wave cycle^64^:

18 $${e}_{{\rm{tot}}}={e}_{{\rm{m}}}+{e}_{{\rm{k}}}+{e}_{{\rm{u}}}$$

19 $$e_{\mathrm{tot}}=\frac{{{\bf{B}}}_{1}\cdot {{\bf{B}}}_{1}^{* }}{4{\mu }_{0}}+\frac{{\rho }_{0}{{\bf{v}}}_{1}\cdot {{\bf{v}}}_{1}^{* }}{4}+\frac{{p}_{1}{p}_{1}^{* }}{4{\rho }_{0}{v}_{\mathrm{s}}^{2}}$$

where the asterisk denotes the complex conjugate of a variable. The expressions for internal and external energy densities are given as follows:

20 $${e}_{{\rm{m}},{\rm{i}}}=\frac{{\left|{C}_{1}\right|}^{2}{\rho }_{0i}{v}_{{\mathrm{Ai}}}^{2}{\left({k}_{z}^{2}{v}_{{\mathrm{si}}}^{2}-{\omega }^{2}\right)}^{2}}{4{\omega }^{6}}\left[{J}_{m}^{2}\left({\widetilde{\kappa }}_{\mathrm{i}}r\right)+\frac{{k}_{z}^{2}}{{\widetilde{\kappa }}_{\mathrm{i}}^{4}}{{\mathscr{J}}}_{m}\right]$$

21 $${e}_{{\rm{m}},{\rm{e}}}=\frac{{\left|{C}_{2}\right|}^{2}{\rho }_{0{\mathrm{e}}}{v}_{{\mathrm{Ae}}}^{2}{\left({k}_{z}^{2}{v}_{{\mathrm{se}}}^{2}-{\omega }^{2}\right)}^{2}}{4{\omega }^{6}}\left[{K}_{m}^{2}\left({\kappa }_{\mathrm{e}}r\right)+\frac{{k}_{z}^{2}}{{\kappa }_{\mathrm{e}}^{4}}{{\mathscr{K}}}_{m}\right]$$

22 $${e}_{{\rm{k}},{\rm{i}}}=\frac{{\left|{C}_{1}\right|}^{2}{\rho }_{0{\mathrm{i}}}}{4{\omega }^{4}}\left[{k}_{z}^{2}{v}_{{\mathrm{si}}}^{4}{J}_{m}^{2}\left({\widetilde{\kappa }}_{\mathrm{i}}r\right)+\frac{{\left({k}_{z}^{2}{v}_{{\mathrm{si}}}^{2}-{\omega }^{2}\right)}^{2}}{{\widetilde{\kappa }}_{\mathrm{i}}^{4}}{{\mathscr{J}}}_{m}\right]$$

23 $${e}_{{\rm{k}},{\rm{e}}}=\frac{{\left|{C}_{2}\right|}^{2}{\rho }_{0{\mathrm{e}}}}{4{\omega }^{4}}\left[{k}_{z}^{2}{v}_{{\mathrm{se}}}^{4}{K}_{m}^{2}\left({\kappa }_{\mathrm{e}}r\right)+\frac{{\left({k}_{z}^{2}{v}_{{\mathrm{se}}}^{2}-{\omega }^{2}\right)}^{2}}{{\kappa }_{\mathrm{e}}^{4}}{{\mathscr{K}}}_{m}\right]$$

24 $${e}_{{\rm{u}},{\rm{i}}}=\frac{{\left|{C}_{1}\right|}^{2}{\rho }_{0{\mathrm{i}}}{v}_{{\mathrm{si}}}^{2}{J}_{m}^{2}\left({\widetilde{\kappa }}_{\mathrm{i}}r\right)}{4{\omega }^{2}},{e}_{{\rm{u}},{\rm{e}}}=\frac{{\left|{C}_{2}\right|}^{2}{\rho }_{0{\mathrm{e}}}{v}_{{\mathrm{se}}}^{2}{K}_{m}^{2}\left({\kappa }_{\mathrm{e}}r\right)}{4{\omega }^{2}},$$

25 $$\begin{array}{c}{e}_{{\rm{tot}},{\rm{i}}}=\frac{{\left|{C}_{1}\right|}^{2}{\rho }_{0{\mathrm{i}}}}{4{\omega }^{4}}\left[\frac{\left({k}_{z}^{2}{v}_{{\mathrm{si}}}^{2}+{\omega }^{2}\right){v}_{{\mathrm{si}}}^{2}{\omega }^{2}+{v}_{{\mathrm{Ai}}}^{2}{\left({k}_{z}^{2}{v}_{{\mathrm{si}}}^{2}-{\omega }^{2}\right)}^{2}}{{\omega }^{2}}{J}_{m}^{2}\left({\widetilde{\kappa }}_{\mathrm{i}}r\right)\right.\\ \left.+\frac{{\left({k}_{z}^{2}{v}_{{\mathrm{si}}}^{2}-{\omega }^{2}\right)}^{2}\left({k}_{z}^{2}{v}_{{\mathrm{Ai}}}^{2}+{\omega }^{2}\right)}{{\omega }^{2}{\widetilde{\kappa }}_{\mathrm{i}}^{4}}{{\mathscr{J}}}_{m}\right]\end{array}$$

26 $$\begin{array}{c}{e}_{{\rm{tot}},{\rm{e}}}{\rm{}}=\frac{{\left|{C}_{2}\right|}^{2}{\rho }_{0{\mathrm{e}}}}{4{\omega }^{4}}\left[\frac{\left({k}_{z}^{2}{v}_{{\mathrm{se}}}^{2}+{\omega }^{2}\right){v}_{{\mathrm{se}}}^{2}{\omega }^{2}+{v}_{{\mathrm{Ae}}}^{2}{\left({k}_{z}^{2}{v}_{{\mathrm{se}}}^{2}-{\omega }^{2}\right)}^{2}}{{\omega }^{2}}{K}_{m}^{2}\left({\kappa }_{\mathrm{e}}r\right)\right.\\ {\rm{}}\left.+\frac{{\left({k}_{z}^{2}{v}_{{\mathrm{se}}}^{2}-{\omega }^{2}\right)}^{2}\left({k}_{z}^{2}{v}_{{\mathrm{Ae}}}^{2}+{\omega }^{2}\right)}{{\omega }^{2}{\kappa }_{\mathrm{e}}^{4}}{{\mathscr{K}}}_{m}\right]\end{array}$$

Here two quantities are defined as:

$${{\mathscr{J}}}_{m}=2{m}^{2}\frac{{J}_{m}^{2}\left({\widetilde{\kappa }}_{\mathrm{i}}r\right)}{{r}^{2}}-2m{\widetilde{\kappa }}_{\mathrm{i}}\frac{{J}_{m+1}\left({\widetilde{\kappa }}_{\mathrm{i}}r\right){J}_{m}\left({\widetilde{\kappa }}_{\mathrm{i}}r\right)}{r}+{\widetilde{\kappa }}_{\mathrm{i}}^{2}{J}_{m+1}^{2}\left({\widetilde{\kappa }}_{\mathrm{i}}r\right)$$

$${{\mathscr{K}}}_{m}=2{m}^{2}\frac{{K}_{m}^{2}\left({\kappa }_{\mathrm{e}}r\right)}{{r}^{2}}-2m{\kappa }_{\mathrm{e}}\frac{{K}_{m+1}\left({\kappa }_{\mathrm{e}}r\right){K}_{m}\left({\kappa }_{\mathrm{e}}r\right)}{r}+{\kappa }_{\mathrm{e}}^{2}{K}_{m+1}^{2}\left({\kappa }_{\mathrm{e}}r\right).$$

The energy density flux (*f*_tot_) has two constituents: the Poynting flux (*f*_m_) and thermal energy flux (*f*_t_). They are averaged over one wave cycle and are given as^64^:

27 $${f}_{{\rm{tot}}}={f}_{{\rm{m}}}+{f}_{{\rm{t}}},$$

28 $$f_{\mathrm{tot}}=\frac{1}{2{\mu }_{0}}\mathrm{Re}\{{{\bf{E}}}_{1}\times {{\bf{B}}}_{1}^{\ast }\}+\frac{1}{2}{\rm{Re}}\{{p}_{1}{\bf{v}}_{1}^{\ast }\}.$$

The explicit expressions for energy density fluxes inside and outside the cylinder are given as follows:

29 $${f}_{{\rm{m}},{\rm{i}}}=\frac{{\left|{C}_{1}\right|}^{2}{\rho }_{0{\mathrm{i}}}{v}_{{\mathrm{Ai}}}^{2}{\left({k}_{z}^{2}{v}_{{\mathrm{si}}}^{2}-{\omega }^{2}\right)}^{2}m{J}_{m}^{2}\left({\widetilde{\kappa }}_{\mathrm{i}}r\right)}{2r{\omega }^{5}{\widetilde{\kappa }}_{\mathrm{i}}^{2}}{\mathbf{\hat{\varphi}}}+\frac{{\left|{C}_{1}\right|}^{2}{\rho }_{0{\mathrm{i}}}{v}_{{\mathrm{Ai}}}^{2}{k}_{z}{\left({k}_{z}^{2}{v}_{{\mathrm{si}}}^{2}-{\omega }^{2}\right)}^{2}{{\mathscr{J}}}_{m}}{2{\omega }^{5}{\widetilde{\kappa }}_{\mathrm{i}}^{4}}\hat{\bf{z}},$$

30 $$\begin{array}{rcl}{f}_{{\rm{m}},{\rm{e}}} &=& -\frac{{\left|{C}_{2}\right|}^{2}{\rho }_{0{\mathrm{e}}}{v}_{{\mathrm{Ae}}}^{2}{\left({k}_{z}^{2}{v}_{{\mathrm{se}}}^{2}-{\omega }^{2}\right)}^{2}m{K}_{m}^{2}\left({\kappa }_{\mathrm{e}}r\right)}{2r{\omega }^{5}{\kappa }_{\mathrm{e}}^{2}}{\mathbf{\hat{\varphi}}} \\ &&+\frac{{\left|{C}_{2}\right|}^{2}{\rho }_{0{\mathrm{e}}}{v}_{{\mathrm{Ae}}}^{2}{k}_{z}{\left({k}_{z}^{2}{v}_{{\mathrm{se}}}^{2}-{\omega }^{2}\right)}^{2}{{\mathscr{K}}}_{m}}{2{\omega }^{5}{\kappa }_{\mathrm{e}}^{4}}\hat{\bf{z}},\end{array}$$

31 $${f}_{{\rm{t}},{\rm{i}}}=-\frac{{|{C}_{1}|}^{2}{\rho }_{0{\mathrm{i}}}{v}_{\mathrm{si}}^{2}({k}_{z}^{2}{v}_{\mathrm{si}}^{2}-{\omega }^{2})m{J}_{m}^{2}({\tilde{\kappa }}_{\mathrm{i}}r)}{2r{\omega }^{3}{\tilde{\kappa }}_{\mathrm{i}}^{2}}{\mathbf{\hat{\varphi}}}+\frac{{|{C}_{1}|}^{2}{\rho }_{0{\mathrm{i}}}{v}_{\mathrm{si}}^{4}{k}_{z}{J}_{m}^{2}({\tilde{\kappa }}_{\mathrm{i}}r)}{2{\omega }^{3}}\hat{\bf{z}},$$

32 $${f}_{{\rm{t}},{\rm{e}}}=\frac{{\left|{C}_{2}\right|}^{2}{\rho }_{0{\mathrm{e}}}{v}_{{\mathrm{se}}}^{2}\left({k}_{z}^{2}{v}_{{\mathrm{se}}}^{2}-{\omega }^{2}\right)m{K}_{m}^{2}\left({\kappa }_{\mathrm{e}}r\right)}{2r{\omega }^{3}{\kappa }_{\mathrm{e}}^{2}}{\mathbf{\hat{\varphi}}}+\frac{{\left|{C}_{2}\right|}^{2}{\rho }_{0{\mathrm{e}}}{v}_{{\mathrm{se}}}^{4}{k}_{z}{K}_{m}^{2}\left({\kappa }_{\mathrm{e}}r\right)}{2{\omega }^{3}}\hat{\bf{z}},$$

33 $$\begin{array}{l}{f}_{{\rm{tot}},{\rm{i}}}=-\frac{{\left|{C}_{1}\right|}^{2}{\rho }_{0{\mathrm{i}}}{\left({k}_{z}^{2}{v}_{{\mathrm{si}}}^{2}-{\omega }^{2}\right)}^{2}\left({k}_{z}^{2}{v}_{{\mathrm{Ai}}}^{2}-{\omega }^{2}\right)m{J}_{m}^{2}\left({\widetilde{\kappa }}_{\mathrm{i}}r\right)}{2r{\omega }^{5}{\widetilde{\kappa }}_{\mathrm{i}}^{4}}{\mathbf{\hat{\varphi}}}\\\qquad\ \ \ +\frac{{\left|{C}_{1}\right|}^{2}{\rho }_{0{\mathrm{i}}}{k}_{z}\left[{v}_{{\mathrm{Ai}}}^{2}{\left({k}_{z}^{2}{v}_{{\mathrm{si}}}^{2}-{\omega }^{2}\right)}^{2}{{\mathscr{J}}}_{m}+{\widetilde{\kappa }}_{\mathrm{i}}^{4}{v}_{{\mathrm{si}}}^{4}{\omega }^{2}{J}_{m}^{2}\left({\widetilde{\kappa }}_{\mathrm{i}}r\right)\right]}{2{\omega }^{5}{\widetilde{\kappa }}_{\mathrm{i}}^{4}}\hat{{\bf{m}}},\end{array}$$

34 $$\begin{array}{l}{f}_{{\rm{tot}},{\rm{e}}}=-\frac{{\left|{C}_{2}\right|}^{2}{\rho }_{0{\mathrm{e}}}{\left({k}_{z}^{2}{v}_{{\mathrm{se}}}^{2}-{\omega }^{2}\right)}^{2}\left({k}_{z}^{2}{v}_{{\mathrm{Ae}}}^{2}-{\omega }^{2}\right)m{K}_{m}^{2}\left({\kappa }_{\mathrm{e}}r\right)}{2r{\omega }^{5}{\kappa }_{e}^{4}}{\mathbf{\hat{\varphi}}}\\\qquad\ \ \ +\frac{{\left|{C}_{2}\right|}^{2}{\rho }_{0{\mathrm{e}}}{k}_{z}\left[{v}_{{\mathrm{Ae}}}^{2}{\left({k}_{z}^{2}{v}_{{\mathrm{se}}}^{2}-{\omega }^{2}\right)}^{2}{{\mathscr{K}}}_{m}+{\kappa }_{\mathrm{e}}^{4}{v}_{{\mathrm{se}}}^{4}{\omega }^{2}{K}_{m}^{2}\left({\kappa }_{\mathrm{e}}r\right)\right]}{2{\omega }^{5}{\kappa }_{\mathrm{e}}^{4}}\hat{\bf{z}},\end{array}$$

Where ${\mathbf{\hat{\varphi}}}$ and $\hat{\bf{z}}$ are unit vectors along the *φ* and *z* directions.

The formulae for energy densities (equations (20)–(26)) and energy density fluxes (equations (29)–(34)) are position-dependent. We therefore integrated the equations over the *r*–*φ* plane to calculate the integrated energy densities (*E*_m_, *E*_k_ and *E*_u_) and integrated energy density fluxes (*F*_m_ and *F*_t_). The following formulae applicable for $x\in {{\mathbb{R}}}^{+}$ were used to obtain integrals of modified Bessel functions^65^:

35 $$\int x{J}_{m}^{2}\left(x\right){{\mathrm{d}}x}=\frac{{x}^{2}}{2}\left({J}_{m}^{2}\left(x\right)-{J}_{m-1}\left(x\right){J}_{m+1}\left(x\right)\right)+C,$$

36 $$\int x{K}_{m}^{2}(x){{\mathrm{d}}x}=\frac{{x}^{2}}{2}\left({K}_{m}^{2}(x)-{K}_{m-1}(x){K}_{m+1}(x)\right)+C,$$

37 $$\int \frac{{J}_{m}^{2}(x)}{x}{{\mathrm{d}}x}=-\frac{{J}_{0}^{2}(x)+{J}_{m}^{2}(x)}{2m}-H(m-2)\frac{1}{m}\mathop{\sum }\limits_{k=1}^{m-1}{J}_{k}^{2}(x)+C,$$

38 $$\int \frac{{K}_{m}^{2}(x)}{x}{{\mathrm{d}}x}=\frac{{(-1)}^{m+1}{K}_{0}^{2}(x)-{K}_{m}^{2}(x)}{2m}-H(m-2)\frac{1}{m}\mathop{\sum }\limits_{k=1}^{m-1}{(-1)}^{m-k}{K}_{k}^{2}(x)+C,$$

39 $$\int {J}_{m+1}(x){J}_{m}(x){{\mathrm{d}}x}=\frac{1}{2}-\frac{1}{2}{J}_{0}^{2}(x)-H(m-1)\mathop{\sum }\limits_{k=1}^{m}{J}_{k}^{2}(x)+C,$$

40 $$\int {K}_{m+1}(x){K}_{m}(x){{\mathrm{d}}x}={(-1)}^{m}\left(\frac{1}{2}-\frac{1}{2}{K}_{0}^{2}(x)-H(m-1)\mathop{\sum }\limits_{k=1}^{m}{(-1)}^{k}{K}_{k}^{2}(x)\right)+C$$

where *C* is the integration constant, *H*(*x*) is a Heaviside function that equals 1 if *x* ≥ 0 and 0 otherwise. Equations (35), (36), (39) and (40) are valid for *m* as natural numbers whereas equations (37) and (38) are valid for *m* as non-zero natural numbers.

As the sausage (*m* = 0) and the kink (*m* = 1) modes are the most commonly observed in the solar atmosphere, they have been studied intensively for coronal heating and seismology^13,66,67^. If we consider the sausage mode (axisymmetric case), the energy density and fluxes are obtained by inserting *m* = 0 in all the preceding formulae. This case has been extensively discussed in ref. ^68^.

In this study, we only considered the kink mode wave with the azimuthal mode number *m* = 1. The integrated energy densities are then given by:

41 $${E}_{{\rm{m}},{\rm{i}}}=\frac{\uppi {\left|{C}_{1}\right|}^{2}{\rho }_{0{\mathrm{i}}}{v}_{{\mathrm{Ai}}}^{2}{\left({k}_{z}^{2}{v}_{{\mathrm{si}}}^{2}-{\omega }^{2}\right)}^{2}}{2{\omega }^{6}}\cdot \left\{{A}_{J}+\frac{{k}_{z}^{2}}{{\widetilde{\kappa }}_{\mathrm{i}}^{4}}{B}_{J}\right\},$$

42 $${E}_{{\rm{m}},{\rm{e}}}=\frac{\uppi {\left|{C}_{2}\right|}^{2}{\rho }_{0{\mathrm{e}}}{v}_{{\mathrm{Ae}}}^{2}{\left({k}_{z}^{2}{v}_{{\mathrm{se}}}^{2}-{\omega }^{2}\right)}^{2}}{2{\omega }^{6}}\cdot \left\{{A}_{K}+\frac{{k}_{z}^{2}}{{\kappa }_{\mathrm{e}}^{4}}{B}_{K}\right\},$$

43 $${E}_{{\rm{k}},{\rm{i}}}=\frac{\uppi {\left|{C}_{1}\right|}^{2}{\rho }_{0{\mathrm{i}}}}{2{\omega }^{4}}\cdot \left\{{k}_{z}^{2}{v}_{{\mathrm{si}}}^{4}{A}_{J}+\frac{{\left({k}_{z}^{2}{v}_{{\mathrm{si}}}^{2}-{\omega }^{2}\right)}^{2}}{{\widetilde{\kappa }}_{\mathrm{i}}^{4}}{B}_{J}\right\},$$

44 $${E}_{{\rm{k}},{\rm{e}}}=\frac{\uppi {\left|{C}_{2}\right|}^{2}{\rho }_{0{\mathrm{e}}}}{2{\omega }^{4}}\cdot \left\{{k}_{z}^{2}{v}_{{\mathrm{se}}}^{4}{A}_{K}+\frac{{\left({k}_{z}^{2}{v}_{{\mathrm{se}}}^{2}-{\omega }^{2}\right)}^{2}}{{\kappa }_{\mathrm{e}}^{4}}{B}_{K}\right\},$$

45 $${E}_{{\rm{u}},{\rm{i}}}=\frac{\uppi {\left|{C}_{1}\right|}^{2}{\rho }_{0{\mathrm{i}}}{v}_{{\mathrm{si}}}^{2}{A}_{J}}{2{\omega }^{2}},{E}_{{\rm{u}},{\rm{e}}}=\frac{\uppi {\left|{C}_{2}\right|}^{2}{\rho }_{0{\mathrm{e}}}{v}_{{\mathrm{se}}}^{2}{A}_{K}}{2{\omega }^{2}},$$

46 $$\begin{array}{l}{E}_{{\rm{tot}},{\rm{i}}}=\frac{\uppi {\left|{C}_{1}\right|}^{2}{\rho }_{0{\mathrm{i}}}}{2{\omega }^{4}}\left\{\frac{\left({k}_{z}^{2}{v}_{{\mathrm{si}}}^{2}+{\omega }^{2}\right){v}_{{\mathrm{si}}}^{2}{\omega }^{2}+{v}_{{\mathrm{Ai}}}^{2}{\left({k}_{z}^{2}{v}_{{\mathrm{si}}}^{2}-{\omega }^{2}\right)}^{2}}{{\omega }^{2}}{A}_{J}\right.\\\qquad\ \ \ \left.+\frac{{\left({k}_{z}^{2}{v}_{{\mathrm{si}}}^{2}-{\omega }^{2}\right)}^{2}\left({k}_{z}^{2}{v}_{{\mathrm{Ai}}}^{2}+{\omega }^{2}\right)}{{\omega }^{2}{\widetilde{\kappa }}_{\mathrm{i}}^{4}}{B}_{J}\right\},\end{array}$$

47 $$\begin{array}{l}{E}_{{\rm{tot}},{\rm{e}}}=\frac{\uppi {\left|{C}_{2}\right|}^{2}{\rho }_{0{\mathrm{e}}}}{2{\omega }^{4}}\left\{\frac{\left({k}_{z}^{2}{v}_{{\mathrm{se}}}^{2}+{\omega }^{2}\right){v}_{{\mathrm{se}}}^{2}{\omega }^{2}+{v}_{{\mathrm{Ae}}}^{2}{\left({k}_{z}^{2}{v}_{{\mathrm{se}}}^{2}-{\omega }^{2}\right)}^{2}}{{\omega }^{2}}{A}_{K}\right.\\ \qquad\ \ \ \left.+\frac{{\left({k}_{z}^{2}{v}_{{\mathrm{se}}}^{2}-{\omega }^{2}\right)}^{2}\left({k}_{z}^{2}{v}_{{\mathrm{Ae}}}^{2}+{\omega }^{2}\right)}{{\omega }^{2}{\kappa }_{\mathrm{e}}^{4}}{B}_{K}\right\}.\end{array}$$

The integrated energy density fluxes are given by:

48 $${F}_{{\rm{m}},{\rm{i}}}=\frac{\uppi {\left|{C}_{1}\right|}^{2}{\rho }_{0{\mathrm{i}}}{v}_{{\mathrm{Ai}}}^{2}{\left({k}_{z}^{2}{v}_{{\mathrm{si}}}^{2}-{\omega }^{2}\right)}^{2}{C}_{J}}{{\omega }^{5}{\widetilde{\kappa }}_{\mathrm{i}}^{2}}{\mathbf{\hat{\varphi}}}+\frac{\uppi {\left|{C}_{1}\right|}^{2}{\rho }_{0{\mathrm{i}}}{v}_{{\mathrm{Ai}}}^{2}{k}_{z}{\left({k}_{z}^{2}{v}_{{\mathrm{si}}}^{2}-{\omega }^{2}\right)}^{2}{B}_{J}}{{\omega }^{5}{\widetilde{\kappa }}_{i}^{4}}\hat{\bf{z}},$$

49 $${F}_{{\rm{m}},{\rm{e}}}=-\frac{\uppi {\left|{C}_{2}\right|}^{2}{\rho }_{0{\mathrm{e}}}{v}_{{\mathrm{Ae}}}^{2}{\left({k}_{z}^{2}{v}_{{\mathrm{se}}}^{2}-{\omega }^{2}\right)}^{2}{C}_{K}}{{\omega }^{5}{\kappa }_{\mathrm{e}}^{2}}{\mathbf{\hat{\varphi}}}+\frac{\uppi {\left|{C}_{2}\right|}^{2}{\rho }_{0{\mathrm{e}}}{v}_{{\mathrm{Ae}}}^{2}{k}_{z}{\left({k}_{z}^{2}{v}_{{\mathrm{se}}}^{2}-{\omega }^{2}\right)}^{2}{B}_{K}}{{\omega }^{5}{\kappa }_{\mathrm{e}}^{4}}\hat{\bf{z}},$$

50 $${F}_{{\rm{t}},{\rm{i}}}=-\frac{\uppi {\left|{C}_{1}\right|}^{2}{\rho }_{0{\mathrm{i}}}{v}_{{\mathrm{si}}}^{2}\left({k}_{z}^{2}{v}_{{\mathrm{si}}}^{2}-{\omega }^{2}\right){C}_{J}}{{\omega }^{3}{\widetilde{\kappa }}_{\mathrm{i}}^{2}}{\mathbf{\hat{\varphi}}}+\frac{\uppi {\left|{C}_{1}\right|}^{2}{\rho }_{0{\mathrm{i}}}{v}_{{\mathrm{si}}}^{4}{k}_{z}{A}_{J}}{{\omega }^{3}}\hat{\bf{z}},$$

51 $${F}_{{\rm{t}},{\rm{e}}}=\frac{\uppi {\left|{C}_{2}\right|}^{2}{\rho }_{0{\mathrm{e}}}{v}_{{\mathrm{se}}}^{2}\left({k}_{z}^{2}{v}_{{\mathrm{se}}}^{2}-{\omega }^{2}\right){C}_{K}}{{\omega }^{3}{\kappa }_{\mathrm{e}}^{2}}{\mathbf{\hat{\varphi}}}+\frac{\uppi {\left|{C}_{2}\right|}^{2}{\rho }_{0{\mathrm{e}}}{v}_{{\mathrm{se}}}^{4}{k}_{z}{A}_{K}}{{\omega }^{3}}\hat{\bf{z}},$$

52 $$\begin{array}{c}{F}_{{\rm{tot}},{\rm{i}}}=-\frac{\uppi {\left|{C}_{1}\right|}^{2}{\rho }_{0{\mathrm{i}}}{\left({k}_{z}^{2}{v}_{{\mathrm{si}}}^{2}-{\omega }^{2}\right)}^{2}\left({k}_{z}^{2}{v}_{{\mathrm{Ai}}}^{2}-{\omega }^{2}\right){C}_{J}}{{\omega }^{5}{\widetilde{\kappa }}_{\mathrm{i}}^{4}}{\mathbf{\hat{\varphi}}}\\ +\frac{\uppi {\left|{C}_{1}\right|}^{2}{\rho }_{0{\mathrm{i}}}{k}_{z}\left[{v}_{{\mathrm{Ai}}}^{2}{\left({k}_{z}^{2}{v}_{{\mathrm{si}}}^{2}-{\omega }^{2}\right)}^{2}{B}_{J}+{\widetilde{\kappa }}_{\mathrm{i}}^{4}{v}_{{\mathrm{si}}}^{4}{\omega }^{2}{A}_{J}\right]}{{\omega }^{5}{\widetilde{\kappa }}_{\mathrm{i}}^{4}}\hat{\bf{z}},\end{array}$$

53 $$\begin{array}{c}{F}_{{\rm{tot}},{\rm{e}}}=-\frac{\uppi {\left|{C}_{2}\right|}^{2}{\rho }_{0{\mathrm{e}}}{\left({k}_{z}^{2}{v}_{{\mathrm{se}}}^{2}-{\omega }^{2}\right)}^{2}\left({k}_{z}^{2}{v}_{{\mathrm{Ae}}}^{2}-{\omega }^{2}\right){C}_{K}}{{\omega }^{5}{\kappa }_{\mathrm{e}}^{4}}{\mathbf{\hat{\varphi}}}\\ +\frac{\uppi {\left|{C}_{2}\right|}^{2}{\rho }_{0{\mathrm{e}}}{k}_{z}\left[{v}_{{\mathrm{Ae}}}^{2}{\left({k}_{z}^{2}{v}_{{\mathrm{se}}}^{2}-{\omega }^{2}\right)}^{2}{B}_{K}+{\kappa }_{e}^{4}{v}_{{\mathrm{se}}}^{4}{\omega }^{2}{A}_{K}\right]}{{\omega }^{5}{\kappa }_{\mathrm{e}}^{4}}\hat{\bf{z}}.\end{array}$$

Here the parameters are defined as follows,

$$\begin{array}{rcl}{A}_{J} & = & \frac{{a}^{2}}{2}[{J}_{1}^{2}({\tilde{\kappa }}_{\mathrm{i}}a)-{J}_{0}({\tilde{\kappa }}_{\mathrm{i}}a){J}_{2}({\tilde{\kappa }}_{\mathrm{i}}a)]\\ {B}_{J} & = & 2+\frac{({\tilde{\kappa }}_{\mathrm{i}}^{2}{a}^{2}-10){J}_{1}^{2}({\tilde{\kappa }}_{\mathrm{i}}a)}{2}+\frac{({\tilde{\kappa }}_{\mathrm{i}}^{2}{a}^{2}-4){J}_{0}^{2}({\tilde{\kappa }}_{\mathrm{i}}a)}{2},\\ {C}_{J} & = & {\displaystyle\int }_{0}^{a}{J}_{1}^{2}({\tilde{\kappa }}_{\mathrm{i}}r){\mathrm{d}}r,\end{array}$$

$$\begin{array}{c}{A}_{K}=-\frac{{a}^{2}}{2}[{K}_{1}^{2}({\kappa }_{\mathrm{e}}a)-{K}_{0}({\kappa }_{\mathrm{e}}a){K}_{2}({\kappa }_{\mathrm{e}}a)],\\ {B}_{K}={K}_{1}^{2}({\kappa }_{\mathrm{e}}a)[1+\frac{{\kappa }_{\mathrm{e}}^{2}{a}^{2}}{2}]-\frac{{K}_{0}^{2}({\kappa }_{\mathrm{e}}a){\kappa }_{\mathrm{e}}^{2}{a}^{2}}{2}\\ {C}_{K}={\displaystyle\int }_{a}^{+\infty }{K}_{1}^{2}({\kappa }_{\mathrm{e}}r){\mathrm{d}}r.\end{array}$$

*C*_*J*_ and *C*_*K*_ must be evaluated numerically. We first validated equations (41)–(47) and (48)–(53) of a pressureless plasma in the thin tube limit, and have recovered the formulae as given in ref. ^69^.

#### Energy flux of the fibril oscillations

We estimated the energy density and flux carried by the transversely oscillating fibrils by assuming that the fibril oscillation is an eigenmode of an infinite plasma cylinder. We used the formulae for energy density and fluxes derived in a magnetized plasma cylinder and the plasma parameters given by the Stokes inversion (Supplementary Table 2).

For the plasma displacement amplitude, we used the average value of the observed oscillation amplitude, about 52.8 km. The radius of the fibril was set to 500 km and the oscillating period was set to 240 s in accordance with the observations. The longitudinal wavenumber *k*_*z*_ was calculated with the dispersion relation for the body mode given by ref. ^62^. This yielded *k*_*z*_*a* = 1.43.

For the magnetic field inside and outside the fibril, we respectively took values of 2,151 G and 2,465 G, which corresponded to *B*_0_ − δ*B* and *B*_0_ + δ*B* at an optical depth of log *τ* = 0; see Supplementary Table 2. The magnetic field strength was averaged over the core of the umbra and accounted for the spatial variation across the umbra and height stratification at the chromosphere, where the fibril oscillations were detected. We expected that the fibrils would be denser and cooler than the ambient umbral plasma, so we used 4,589 K and 5,492 K for fibril and umbral temperatures, respectively, where densities were 6.33 × 10^−10^ kg m^−3^ and 3.72 × 10^−10^ kg m^−3^. These values corresponded to *T*_0_ − 3δ*T* and *T*_0_ + 3δ*T* for temperature and *ρ* + δ*ρ* and *ρ* − δ*ρ* for density. The thermal pressures inside and outside the fibril were calculated with the state equation of an ideal gas, yielding 23,978 N m^−2^ and 18,249 N m^−2^, respectively. To ensure a stable fibril structure, we adjusted the external thermal pressure to 18,211 N m^−2^, so that force balance was maintained in the radial direction.

We then proceeded to calculate the energy densities and fluxes given by the oscillating fibrils. The fibril occupied about 29–33% of the umbral area, so we took a filling factor of 31% to calculate the energy contents. The theoretical considerations are given by ref. ^70^. The total energy flux directed upwards was estimated to be 7.52 × 10^6^ W m^−2^; this is about two orders of magnitude stronger than the energy flux required for active-region plasma heating^42^. The energy densities and directional energy fluxes are given in Supplementary Table 3.

### Reporting summary

Further information on research design is available in the Nature Portfolio Reporting Summary linked to this article.

### Supplementary information

Supplementary Information Supplementary Figs. 1 and 2 and Tables 1–3.

Reporting Summary

Supplementary Video 1 A video showing the high-resolution observations of transverse motion in the sunspot. This video is associated with Fig. 1.

Supplementary Video 2 A video showing the transversely oscillating fibril in the two-fluid MHD simulation. This video is associated with Fig. 2.

## Data Availability

Observational data used in this study were obtained from the Big Bear Solar Observatory (https://www.bbso.njit.edu/) and the SDO mission (https://sdo.gsfc.nasa.gov/) and are publicly available. The simulation and analytical data are available from the corresponding author (yuanding@hit.edu.cn).

## Code availability

This research used self-developed Python code for data calibration and analysis. A part of the data analysis was done with SolarSoft (https://www.lmsal.com/solarsoft/) based on the Interactive Data Language. Codes and tools are available upon request from the corresponding author (yuanding@hit.edu.cn). The Stokes inversion code, SIR, is publicly available at https://github.com/cdiazbas/SIRcode. The two-fluid MHD modelling code, JOANNA, is available at http://kft.umcs.lublin.pl/dwojcik/about-the-code/.

## References

[1] Bahauddin SM, Bradshaw SJ, Winebarger AR (2021). The origin of reconnection-mediated transient brightenings in the solar transition region. Nat. Astron..

[2] Cargill PJ, Klimchuk JA (2004). Nanoflare heating of the corona revisited. Astrophys. J..

[3] Jafari A, Vishniac ET, Xu S (2021). Nanoflare theory revisited. Astrophys. J..

[4] Srivastava AK (2019). On the observations of rapid forced reconnection in the solar corona. Astrophys. J..

[5] Su Y (2013). Imaging coronal magnetic-field reconnection in a solar flare. Nat. Phys..

[6] Xue Z (2016). Observing the release of twist by magnetic reconnection in a solar filament eruption. Nat. Commun..

[7] McIntosh SW (2011). Alfvénic waves with sufficient energy to power the quiet solar corona and fast solar wind. Nature.

[8] Srivastava AK (2017). High-frequency torsional Alfvén waves as an energy source for coronal heating. Sci. Repo..

[9] De Pontieu B (2007). Chromospheric Alfvénic waves strong enough to power the solar wind. Science.

[10] Srivastava AK (2018). Confined pseudo-shocks as an energy source for the active solar corona. Nat. Astron..

[11] Grant SDT (2018). Alfvén wave dissipation in the solar chromosphere. Nat. Phys..

[12] Srivastava AK (2021). Chromospheric heating by magnetohydrodynamic waves and instabilities. J. Geophys. Res. Space Phys..

[13] Van Doorsselaere T (2020). Coronal heating by MHD waves. Space Sci. Rev..

[14] Banerjee D, Gupta GR, Teriaca L (2011). Propagating MHD waves in coronal holes. Space Sci. Rev..

[15] Banerjee D (2021). Magnetohydrodynamic waves in open coronal structures. Space Sci. Rev..

[16] Wang T (2021). Slow-mode magnetoacoustic waves in coronal loops. Space Sci. Rev..

[17] Alfvén H (1947). Magneto hydrodynamic waves, and the heating of the solar corona. Mon. Not. R. Astron. Soc..

[18] Morton RJ (2012). Observations of ubiquitous compressive waves in the Sun’s chromosphere. Nat. Commun..

[19] Grant SDT (2015). Wave damping observed in upwardly propagating sausage-mode oscillations contained within a magnetic pore. Astrophys. J..

[20] McIntosh SW, De Pontieu B (2012). Estimating the ‘dark’ energy content of the solar corona. Astrophys. J..

[21] Pant V, Magyar N, Van Doorsselaere T, Morton RJ (2019). Investigating ‘dark’ energy in the solar corona using forward modeling of MHD waves. Astrophys. J..

[22] Cao W (2010). Scientific instrumentation for the 1.6 m New Solar Telescope in Big Bear. Astron. Nachr..

[23] Goode, P. R. & Cao, W. The 1.6 m off-axis New Solar Telescope (NST) in Big Bear. In *Groundbased and Airborne Telescopes IV* Conference Series Vol. 8444 (eds Stepp, L. M. et al.) 844403 (SPIE, 2012).

[24] Wöger F, von der Lühe O (2007). Field dependent amplitude calibration of adaptive optics supported solar speckle imaging. Adapt. Optics.

[25] Leenaarts J, Carlsson M, Rouppe van der Voort L (2012). The formation of the H*α* line in the solar chromosphere. Astrophys. J..

[26] Jess DB, Reznikova VE, Doorsselaere TV, Keys PH, Mackay DH (2013). The influence of the magnetic field on running penumbral waves in the solar chromosphere. Astrophys. J..

[27] Su JT (2016). Observations of oppositely directed umbral wavefronts rotating in sunspots obtained from the New Solar Telescope of BBSO. Astrophys. J..

[28] Socas-Navarro H, McIntosh SW, Centeno R, de Wijn AG, Lites BW (2009). Direct imaging of fine structure in the chromosphere of a sunspot umbra. Astrophys. J..

[29] Socas-Navarro H, Trujillo Bueno J, Ruiz Cobo B (2000). Anomalous circular polarization profiles in sunspot chromospheres. Astrophys. J..

[30] Socas-Navarro H, Trujillo Bueno J, Ruiz Cobo B (2000). Anomalous polarization profiles in sunspots: possible origin of umbral flashes. Science.

[31] Borrero JM, Ichimoto K (2011). Magnetic structure of sunspots. Living Rev. Solar Phys..

[32] Khomenko E, Collados M (2015). Oscillations and waves in sunspots. Living Rev. Solar Phys..

[33] Jess DB (2017). An inside look at sunspot oscillations with higher azimuthal wavenumbers. Astrophys. J..

[34] Yuan D, Van Doorsselaere T (2016). Forward modeling of standing kink modes in coronal loops. I. Synthetic views. Astrophys. J. Suppl. Ser..

[35] Yuan D, Van Doorsselaere T (2016). Forward modeling of standing kink modes in coronal loops. II. Applications. Astrophys. J. Suppl. Ser..

[36] Pietarila A, Aznar Cuadrado R, Hirzberger J, Solanki SK (2011). Kink waves in an active region dynamic fibril. Astrophys. J..

[37] Morton RJ, Mooroogen K, Henriques VMJ (2021). Transverse motions in sunspot super-penumbral fibrils. Phil. Trans. R. Soc. A.

[38] Nakariakov VM, Ofman L, Deluca EE, Roberts B, Davila JM (1999). TRACE observation of damped coronal loop oscillations: implications for coronal heating. Science.

[39] Aschwanden MJ, Fletcher L, Schrijver CJ, Alexander D (1999). Coronal loop oscillations observed with the transition region and coronal explorer. Astrophys. J..

[40] Nisticò G, Nakariakov VM, Verwichte E (2013). Decaying and decayless transverse oscillations of a coronal loop. Astron. Astrophys..

[41] Kuźma B, Wójcik D, Murawski K, Yuan D, Poedts S (2020). Numerical simulations of the lower solar atmosphere heating by two-fluid nonlinear Alfvén waves. Astron. Astrophys..

[42] Withbroe GL, Noyes RW (1977). Mass and energy flow in the solar chromosphere and corona. Ann. Rev. Astron. Astrophys..

[43] Kuźma B, Wójcik D, Murawski K (2019). Heating of a quiet region of the solar chromosphere by ion and neutral acoustic waves. Astrophys. J..

[44] Murawski K, Musielak ZE, Wójcik D (2020). 3D numerical simulations of solar quiet chromosphere wave heating. Astrophys. J. Lett..

[45] Kuźma B, Murawski K, Poedts S (2021). 3D numerical simulations of propagating two-fluid, torsional Alfvén waves and heating of a partially ionized solar chromosphere. Mon. Not. R. Astron. Soc..

[46] Anfinogentov SA, Nakariakov VM, Nisticò G (2015). Decayless low-amplitude kink oscillations: a common phenomenon in the solar corona?. Astron. Astrophys..

[47] Yuan D, Nakariakov VM (2012). Measuring the apparent phase speed of propagating EUV disturbances. Astron. Astrophys..

[48] Vögler A (2005). Simulations of magneto-convection in the solar photosphere. Equations, methods, and results of the MURaM code. Astron. Astrophys..

[49] Kuźma B, Murawski K (2018). Numerical simulations of transverse oscillations of a finely structured solar flux tube. Astrophys. J..

[50] Oliver R, Soler R, Terradas J, Zaqarashvili TV (2016). Dynamics of coronal rain and descending plasma blobs in solar prominences. II. Partially ionized case. Astrophys. J..

[51] Ballester JL, Carbonell M, Soler R, Terradas J (2018). The temporal behaviour of MHD waves in a partially ionized prominence-like plasma: effect of heating and cooling. Astron. Astrophys..

[52] Vranjes J, Krstic PS (2013). Collisions, magnetization, and transport coefficients in the lower solar atmosphere. Astron. Astrophys..

[53] Low BC (1985). Three-dimensional structures of magnetostatic atmospheres. I. Theory. Astrophys. J..

[54] Avrett EH, Loeser R (2008). Models of the solar chromosphere and transition region from SUMER and HRTS observations: formation of the extreme-ultraviolet spectrum of hydrogen, carbon, and oxygen. Astrophys. J. Suppl. Ser..

[55] Cao, W. et al. NIRIS: the second generation Near-Infrared Imaging Spectro-polarimeter for the 1.6 meter New Solar Telescope. In *Second ATST-EAST Meeting: Magnetic Fields from the Photosphere to the Corona* Conference Series Vol. 463 (eds Rimmele, T. R. et al.) 291 (Astronomical Society of the Pacific, 2012).

[56] Cao, W., Yurchyshyn, V., Yang, X., Cho, K.-S. & Wang, H. Supporting Parker Solar Probe mission with Goode Solar Telescope at Big Bear Solar Observatory. In *Ground-based and Airborne Instrumentation for Astronomy IX* Conference Series Vol. 12184 (eds Evans, C. J. et al.) 1218428 (SPIE, 2022).

[57] del Toro Iniesta JC, Ruiz Cobo B (2016). Inversion of the radiative transfer equation for polarized light. Living Rev. Solar Phys..

[58] Ruiz Cobo B, Toro Iniesta JC (1992). Inversion of Stokes profiles. Astrophys. J..

[59] Fontenla JM, Avrett EH, Loeser R (1993). Energy balance in the solar transition region. III. Helium emission in hydrostatic, constant abundance models with diffusion. Astrophys. J..

[60] Trelles Arjona JC, Ruiz Cobo B, Martínez González MJ (2021). Empirical determination of atomic line parameters of the 1.5 µm spectral region. Astron. Astrophys..

[61] Asplund M, Grevesse N, Sauval AJ, Scott P (2009). The chemical composition of the Sun. Ann. Rev. Astron. Astrophys..

[62] Edwin PM, Roberts B (1983). Wave propagation in a magnetic cylinder. Solar Phys..

[63] Ruderman MS, Erdélyi R (2009). Transverse oscillations of coronal loops. Space Sci. Rev..

[64] Walker, A. *Magnetohydrodynamic Waves in Geospace: The Theory of ULF Waves and their Interaction with Energetic Particles in the Solar-Terrestrial Environment* (CRC, 2019).

[65] Abramowitz, M. & Stegun, I. *Handbook of Mathematical Functions: With Formulas, Graphs, and Mathematical Tables* (Dover, 1965).

[66] Nakariakov VM (2021). Kink oscillations of coronal loops. Space Sci. Rev..

[67] Li B (2020). Magnetohydrodynamic fast sausage waves in the solar corona. Space Sci. Rev..

[68] Moreels MG, Van Doorsselaere T, Grant SDT, Jess DB, Goossens M (2015). Energy and energy flux in axisymmetric slow and fast waves. Astron. Astrophys..

[69] Goossens M, Van Doorsselaere T, Soler R, Verth G (2013). Energy content and propagation in transverse solar atmospheric waves. Astrophys. J..

[70] Van Doorsselaere T, Gijsen SE, Andries J, Verth G (2014). Energy propagation by transverse waves in multiple flux tube systems using filling factors. Astrophys. J..

