# Supplementary material for: Transverse oscillations and an energy source in a strongly magnetized sunspot
Source: Nat Astron. 2023 May 25;7(7):856–66. doi: 10.1038/s41550-023-01973-3 (PMC10356614; doi:10.1038/s41550-023-01973-3)
Supplement: Supplementary file 1 — Supplementary Figs. 1 and 2 and Tables 1–3. [file 41550_2023_1973_MOESM1_ESM.pdf]

nature astronomy

Article

https://doi.org/10.1038/s41550-023-01973-3

Transverse oscillations and an energy source in a strongly magnetized sunspot

In the format provided by the authors and unedited

This Supplementary Information file contains:

- **Supplementary Figures**
  - **Supplementary Figure 1**. The sunspot atmosphere used in the simulation.
  - **Supplementary Figure 2**. Parameters of sunspot AR12384 obtained with Stokes inversion.
- **Supplementary Tables**
  - **Supplementary Table 1**. The measured parameters of the oscillating fibril.
  - **Supplementary Table 2**. Parameters of the plasma and magnetic field averaged within the sunspot umbra.
  - **Supplementary Table 3**. Estimation of energy densities and flux carried by fibril oscillations

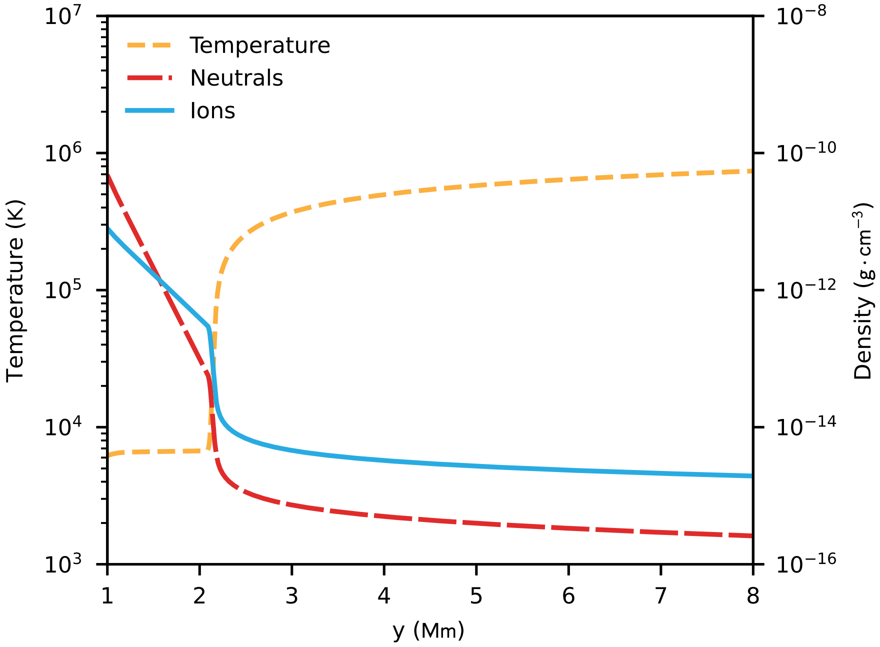

Figure 1: **The sunspot atmosphere used in the simulation.** The variations of temperature, density of ions and neutrals with height.

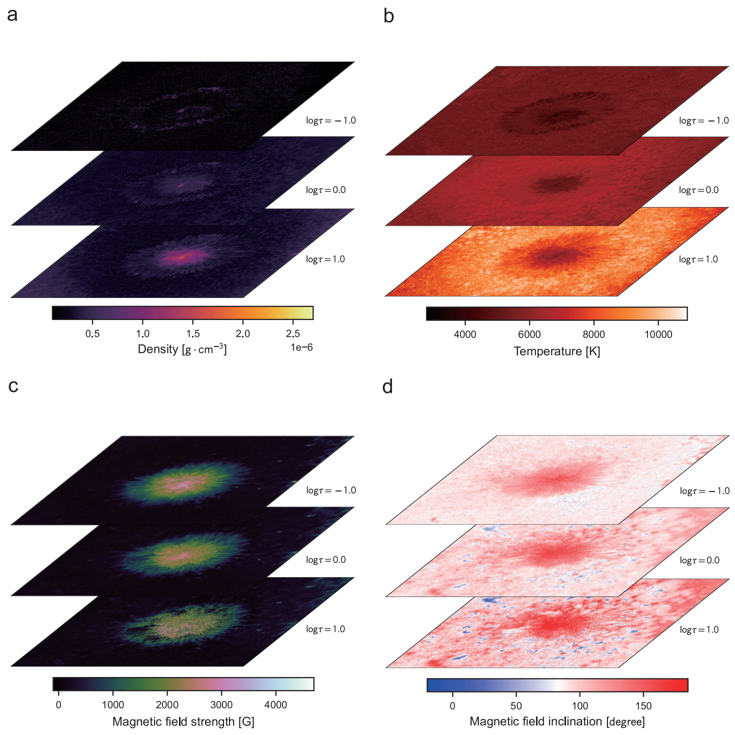

Figure 2: **Parameters of sunspot AR12384 obtained with Stokes inversion. a-d** draws the spatial distributions of plasma density, temperature, magnetic field strength and inclination at three optical depths at $\log\tau = [-1, 0, 1]$.

Supplementary Table 1: The measured parameters of the oscillating fibril

Sample number	Period [s]	Oscillation amplitude [km]	Amplitude in velocity $[\mathrm{km}\cdot\mathrm{s}^{-1}]$
1	173 ± 1.98	39.5 ± 4.48	1.44 ± 0.16
2	281 ± 6.94	50.7 ± 5.05	1.13 ± 0.11
3	219 ± 2.56	62.4 ± 5.37	1.79 ± 0.16
4	265 ± 4.82	45.5 ± 4.86	1.08 ± 0.12
5	304 ± 2.81	54.9 ± 5.93	1.14 ± 0.12
6	275 ± 3.73	40.0 ± 5.87	0.92 ± 0.13
7	191 ± 2.81	43.4 ± 4.76	1.43 ± 0.16
8	200 ± 2.23	59.3 ± 5.88	1.87 ± 0.19
Average value	239	49.5	1.35
Standard deviation	48.6	8.75	0.34

Supplementary Table 2: Parameters of the plasma and magnetic field averaged within the sunspot umbra

Optical depth	Density (10^{-6}g · m^{-3})		Temperature (K)		Magnetic field strength (G)	
$\log \tau$	ρ_0	$\delta\rho$	T_0	δT	B_0	δB
1.0	1.071	0.119	6392	318	2102	384
0.9	0.963	0.100	6404	239	2122	359
0.8	0.878	0.090	6401	185	2142	334
0.7	0.814	0.088	6335	159	2163	310
0.6	0.762	0.091	6227	154	2183	287
0.5	0.715	0.095	6086	166	2204	265
0.4	0.673	0.100	5904	185	2224	243
0.3	0.630	0.107	5723	204	2245	221
0.2	0.587	0.114	5549	217	2266	199
0.1	0.544	0.122	5397	224	2287	177
0.0	0.502	0.130	5265	225	2308	156
-0.1	0.462	0.139	5156	222	2328	140
-0.2	0.424	0.148	5064	216	2349	132
-0.3	0.390	0.157	4985	211	2370	133
-0.4	0.359	0.166	4920	209	2391	143
-0.5	0.332	0.175	4859	212	2412	161
-0.6	0.309	0.183	4798	222	2433	182
-0.7	0.288	0.191	4733	242	2454	205
-0.8	0.270	0.197	4674	272	2475	229
-0.9	0.253	0.200	4622	310	2496	253
-1.0	0.236	0.200	4578	353	2517	278

Supplementary Table 3: Estimation of energy densities and flux carried by fibril oscillations

Kinetic energy density ($J \cdot m^{-3}$)	8.38×10^{-4}
Magnetic energy density ($J \cdot m^{-3}$)	2.19×10^{-4}
Internal energy density ($J \cdot m^{-3}$)	6.14×10^{-4}
Total energy densities ($J \cdot m^{-3}$)	1.67×10^{-3}
Poynting energy density flux (φ-direction, $W \cdot m^{-2}$)	6.48×10^{4}
Poynting energy density flux (z-direction, $W \cdot m^{-2}$)	5.59×10^{6}
Thermal energy density flux components (φ-direction, $W \cdot m^{-2}$)	-6.21×10^{5}
Thermal energy density flux components (z-direction, $W \cdot m^{-2}$)	1.92×10^{6}
Total energy density flux components (φ-direction, $W \cdot m^{-2}$)	-5.56×10^{5}
Total energy density flux components (z-direction, $W \cdot m^{-2}$)	7.52×10^{6}
