# Supplementary material for: Transverse oscillations and an energy source in a strongly magnetized sunspot
Source: Nat Astron. 2023 May 25;7(7):856–66. doi: 10.1038/s41550-023-01973-3 (PMC10356614; doi:10.1038/s41550-023-01973-3)
Supplement: Supplementary file 2 — Reporting Summary [file 41550_2023_1973_MOESM2_ESM.pdf]

Nature Research’s Reporting Summary

(162 words)

Why is the solar corona much hotter than the underlying solar surface, although it is much farther away from the energy source at the sun's core? This is a century-long standing dilemma for physicists. Yuan and his collaborators used the high-resolution observation of the Goode Solar Telescope, the largest solar telescope in the world. They detected the transverse motions within a strongly magnetized sunspot. These waves dragged the kilo-gauss magnetic field laterally, which required a force of about 1000 times more potent than other regions of the sun, and was estimated to carry an enormous amount of energy flux directed outwards towards the overlying atmosphere of the Sun——active region (corona). Only about 0.1 % - 0.01% of the energy flux is sufficient for solar coronal heating, this was demonstrated with state-of-the-art supercomputer simulations. This finding could become transformative knowledge for coronal heating and could be the research highlight for the next-generate solar telescopes.
